# Multiphoton Nanosculpting of Optical Resonant and Nonresonant Microsensors on Fiber Tips

**DOI:** 10.1021/acsami.2c01033

**Published:** 2022-04-12

**Authors:** Jeremiah C. Williams, Hengky Chandrahalim, Joseph S. Suelzer, Nicholas G. Usechak

**Affiliations:** † Department of Electrical and Computer Engineering, Air Force Institute of Technology, Wright−Patterson Air Force Base, Dayton, Ohio 45433, United States; ‡ Sensors Directorate, Air Force Research Laboratory, Wright−Patterson Air Force Base, Dayton, Ohio 45433, United States

**Keywords:** multiphoton polymerization, flow sensors, pressure sensors, 3D nanofabrication, Fabry−Pérot, optical sensors, optical fiber sensors, nanomachining

## Abstract

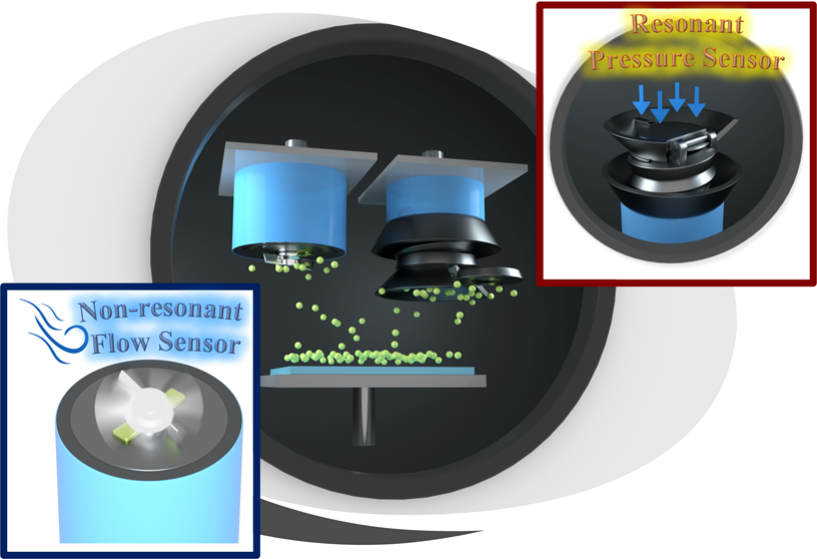

This work presents a multiphoton nanosculpting process that is employed to fabricate three-dimensional (3D) mechanically assisted optical resonant and nonresonant microsensors on fiber tips. The resonant microsensor consists of a complex 3D optical cavity design with submicron resolution and advanced micromechanical features including a hinged, multipositional mirror, a 3D spring body to displace this mirror without deforming it, and adhesive-retaining features for sealing the cavity. These features represent a breakthrough in the integration and fabrication capabilities of micro-optomechanical systems. The demonstrated dynamic optical surface enables directional thin-film deposition onto obscured areas. We leverage the rotation of the dynamically movable mirror to deposit a thin reflective coating onto the inner surfaces of a Fabry–Pérot cavity (FPC) with curved geometry. The reflective coating in conjunction with the dynamically rotatable mirror greatly improves the quality factor of the FPC and enables a new class of highly integrated multipurpose sensor systems. A unique spring body FPC on an optical fiber tip is used to demonstrate pressure sensing with a sensitivity of 38 ± 7 pm/kPa over a range of −80 to 345 kPa. The nonresonant microsensor consists of microblades that spin in response to an incident flow. Light exiting the core of the optical fiber is reflected back into the fiber core at a flow-dependent rate as the blades pass by. The fiber tip flow sensor operates successfully over a range of 9–25 LPM using nitrogen gas and achieves a linear response of 706 ± 43 reflections/LPM over a range of 10.9–12 LPM. The nanostructuring technology presented in this work offers a path forward for utilizing 3D design freedom in micromechanically enhanced optical and optofluidic systems to facilitate versatile processing and advantageous geometries beyond the current state-of-the-art.

## Introduction

High-performance and compact sensors are important for applications such as aircraft, satellites, wireless wearables, and unmanned aerial systems, which have strict size, weight, and power (SWAP) requirements. Reducing the footprint of these sensors while retaining performance liberates valuable system resources. Optical fibers integrated with micro Fabry–Pérot (FP) resonators represent a promising approach to scale down a variety of sensors that are essential in modern engineering systems. Fiber optics offer lightweight, low losses over long distances, and immunity to electromagnetic interference while serving as an integrated waveguide to introduce and interrogate light. The FP resonator delivers a highly sensitive optical response to a variety of environmental stimuli. Notably, microscale FP cavities exhibit both high sensitivities and large operating ranges at common commercial wavelengths and demonstrate high-quality factors (*Q*-factors) with standard reflective coatings.

Optical fibers with integrated FP sensing elements are reported in the literature with a variety of designs, fabrication processes, and applications. Microscale fiber-optic systems have been demonstrated for sensing pressure,^1−18^ temperature,^1,4,6,7,9−13,17,19−34^ refractive index (RI) and gas,^2,19,20,35−37^ magnetic field strength,^38^ airflow,^39−41^ liquid flow vector,^42^ humidity,^43,44^ acoustic pressure,^45,46^ vibration,^47^ and applied force.^48^ All of these exciting applications could be improved by utilizing reflective coatings and curved surfaces, as enabled by the dynamically movable hinged mirror feature demonstrated in this work. Furthermore, we also explored the advantage of this nanofabrication technology to integrate a spring body microchamber that can retain gas or liquid with the FP sensing element. The flexibility of this technology is also exhibited by the creation of a three-dimensional (3D) microturbine flow sensor on a fiber tip.

Various innovative fabrication techniques have been investigated to realize microscale optical cavities. For example, both closed and open cavities have been demonstrated by splicing single-mode fiber (SMF) into short segments of capillary or internally structured fiber.^5,7,19,20,39^ Open cavities have also been created by removing fiber material with a focused ion beam (FIB)^27^ or femtosecond laser.^36^ Curved surfaces have been fashioned with polymer droplets^4,10^ and the electrical arc of a fiber splicer.^19,35^ Several materials with advantageous properties have been integrated with devices to improve performance including poly(vinyl chlorine) (PVC),^16^ silicone rubber,^15^ silicon,^18,26,42^ liquid mercury,^25^ Nafion,^43^ silver film,^14^ poly(vinyl alcohol) (PVA),^44,49^ high-temperature ceramics,^3^ and selectively sintered stainless steel.^23^ Thin silica films have also been demonstrated by precise etching with hydrofluoric acid (HF)^13^ or etching with HF and fusing to an external silica membrane.^12^ Reflective coatings have been integrated directly in various stages of fabrication^3,11,14^ and through microelectromechanical systems (MEMS) adhered to the fiber.^5,6,8,46^

While remarkable, most fabrication methods are restricted to primitive shapes such as two-dimensional surfaces or large bubbles. The deposition of reflective coatings must also be carefully considered throughout the entire fabrication process as internal optical surfaces are often obscured in the final device. Additive manufacturing techniques enable arbitrary 3D features, such as curved mirrors. Two popular methods for additive manufacturing on optical fibers are stereolithography and two-photon polymerization (2PP) nanofabrication. Stereolithography has been used to fabricate an open FP resonator^2^ and a closed FP resonator with an integrated antireflective microstructure.^1^ 2PP nanofabrication offers even greater precision and has been used to demonstrate very challenging 3D features on optical fiber tips with submicron accuracy. Examples include a force-sensitive microgripper,^50^ multilens objectives,^51^ microring resonators,^52^ whispering gallery mode resonators,^53,54^ an inverse-designed metalens,^55^ a microscale anemometer,^40,41^ a microphone,^56^ and FP cavity sensors.^9,17,21,24,34,37,38,44,45,48,57,58^ This work utilizes 2PP nanofabrication to demonstrate a design solution that enables directional thin-film deposition onto a monolithically integrated spring body optical cavity with dynamic mirrors of arbitrary curvature. Moreover, 2PP nanofabrication is also used to produce a nonresonant microturbine flow sensor on an optical fiber.

A mechanically suspended FP resonator with curved surfaces was reported in our previous work,^21^ but the inner optical surface of the cavity was shadowed by the top surface, which prevented reflective coating deposition. Depositing a gold reflective film by sputtering improved the *Q*-factor of the FP resonator, as shown in Figure S1 (Supporting Information). However, the measured spectrum (blue curve in Figure S1b) was consistent with the resonance between the topmost and bottommost optical surfaces of the cavity only, eliminating the optical response of the inner cavity. This work presents monolithically integrated dynamic optomechanical features that address this problem.

**Figure 1:**
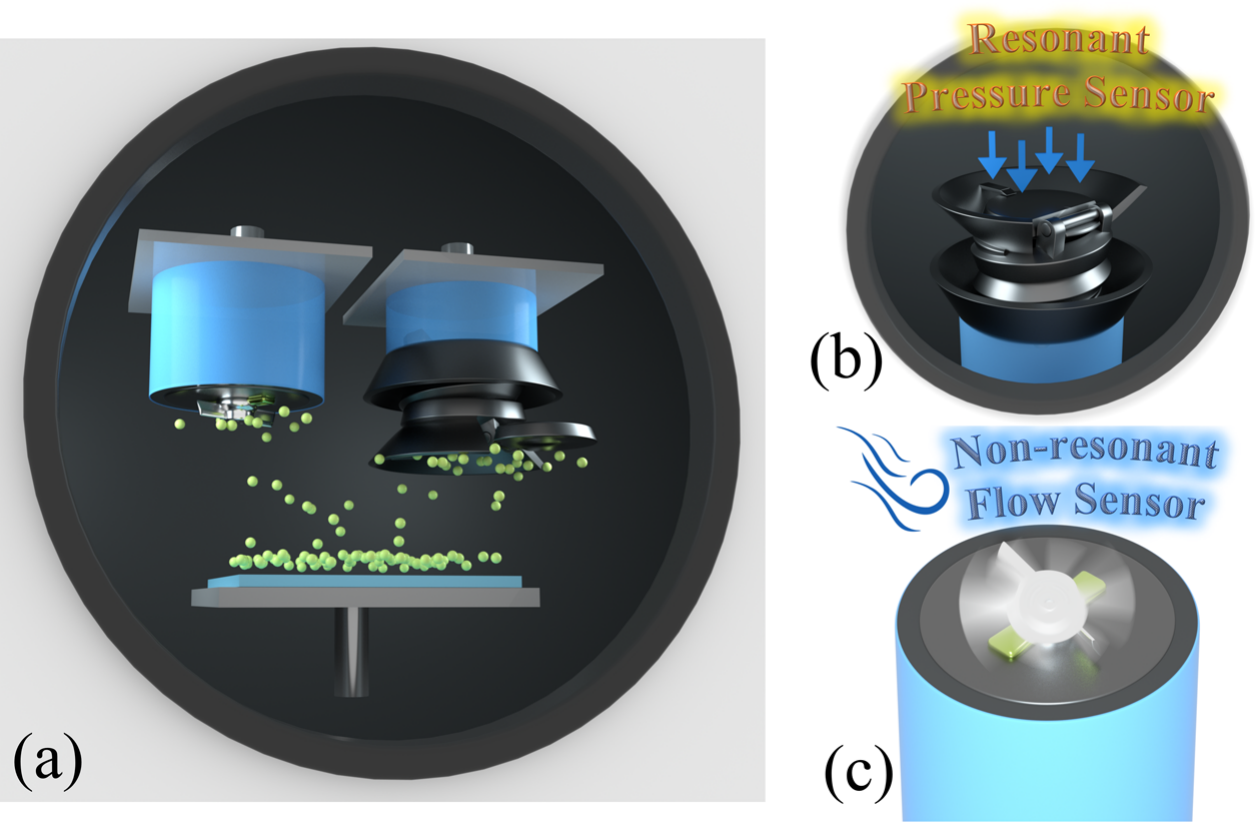
(a) Schematic highlighting the monolithically integrated optomechanical microsystems that enable directional deposition of highly reflective coating onto the targeted surfaces of optical resonant and nonresonant devices using a ubiquitously available magnetron sputtering system. Schematic illustrations of (b) a resonant pressure sensor and (c) a nonresonant flow sensor on an optical fiber tip.

The micromechanically enabled optical systems were fabricated using the highly selective, submicron accuracy of the 2PP nanofabrication method. The design in combination with the multiphoton nanosculpting process facilitates precise, directional thin-film deposition onto all targeted surfaces in the proposed optical resonant and nonresonant microsystems. The implemented mechanical hinge successfully enabled deposition onto the obscured surface, creating an optical resonator that is supported by a spring body microchamber. In addition, an optomechanical nonresonant flow sensor featuring an aerodynamically driven rotor that spins in response to an incident flow is presented. These fiber tip microsystems demonstrated novel pressure and flow sensing, showcasing the feasibility of multipurpose microsensors utilizing this technology. Figure 1a highlights the monolithically integrated optomechanical microsystems that enable directional deposition of a highly reflective thin film onto the targeted surfaces of optical resonant and nonresonant devices using a widely available magnetron sputtering system. For the optical resonant sensor, after the thin reflective coating is sputtered onto the inner surfaces of the cavity, the rotatable mirror is locked into its final position, as shown in Figure 1b. The unique spring body geometry can be used to retain liquid and gas inside the cavity and monitor changes in surrounding pressure over time. For the optical nonresonant flow sensor, a thin reflective coating is sputtered onto the top surface of the microblades to reflect incoming light back into the fiber core at a flow-dependent rate as the blades pass by, as illustrated in Figure 1c.

**Figure 2:**
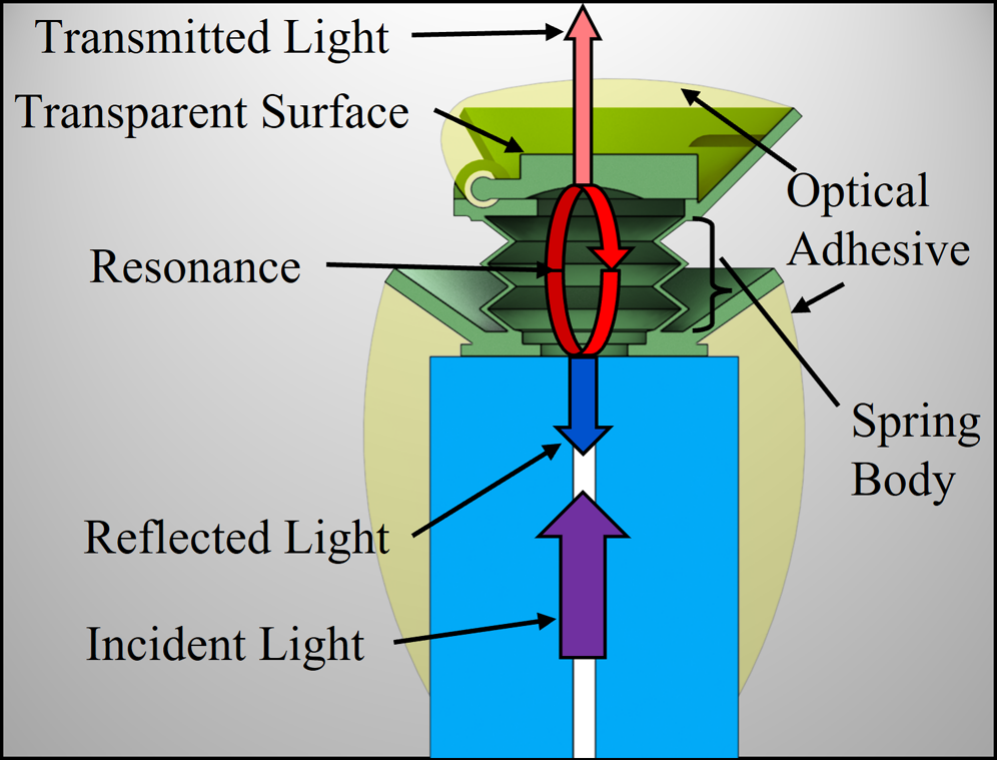
Cross-sectional schematic illustrating the operation of the mechanically enhanced spring body Fabry–Pérot resonator and optical fiber as an integrated waveguide for the device.

The Fabry–Pérot (FP) resonator is a commonly used optical module formed by two parallel, partially reflective mirrors separated by an interstitial medium. Light is introduced propagating perpendicular to the cavity, along the optical axis of the resonator. By tuning the wavelength of the incident light or altering the cavity length, the transmitted light cycles between constructive and destructive interference. The wavelength or cavity is resonant when the constructive interference is largest. A graphical illustration of this interaction in our mechanically enhanced spring body FP resonator is displayed in Figure 2.

The FP resonator has been well modeled in other literature, with a thorough example found in^59^ and relevant relationships briefly highlighted here. The time necessary for light to traverse the resonator cavity and return to its entry point (the round-trip time *t*_RT_) is determined by the length of the cavity *L* and RI of the interstitial medium within the cavity *n* and given by , where *c* is the speed of light in vacuum. This round trip introduces a phase-shift ϕ_RT_ to light of wavelength λ equals to . Resonance repeats after a phase shift equal a multiple *m* of 2π, yielding the resonant wavelength λ_*m*_ to be . Differentiating this equation for λ_*m*_ with respect to *L* and *n* for a single-mode order and taking a linear approximation^60^ yields
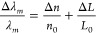
1where *L*_0_, *n*_0_, and λ_*m*_ are the initial values of cavity length, refractive index, and resonant wavelength, respectively. Equation 1 summarizes the sensing mechanism of our devices. Small changes in cavity length and RI produce small changes in the resonant wavelength. One can measure this optical response to observe any environmental phenomenon that affects *L* or *n* over a large range with high sensitivity.

The ideal response of an FP resonator is modeled with an Airy function, which can be analyzed as the sum of individual resonant modes represented with Lorentzian profiles.^59^ Normalized to a maximum transmission value of 1, the ideal Lorentzian shape of an FP resonator’s transmission intensity, *I*_t_, at a single-resonant mode in terms of the incident light’s wavelength is given by
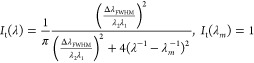
2where Δλ_FWHM_ is the full width at half-maximum (FWHM) of the resonance feature and λ_2_ and λ_1_ are the end points in this bandwidth. Equation 2 represents transmission through the cavity, while our work utilizes reflection from the cavity. Ideally, all light incident to the cavity is either reflected or transmitted such that *I*_T_ = *I*_r_ + *I*_t_, where *I*_T_ is the total incident intensity, *I*_r_ is the reflected intensity, and *I*_t_ is the transmitted intensity. The FWHM is determined by the reflectivity of the optical surfaces of the cavity. For two surfaces of reflectance *R*, the FWHM is related to *R* by −ln (*R*^2^)/2 = 2π*Ln*Δλ_FWHM_/λ_2_λ_1_.^59^ Higher reflectivity yields finer resonant features, which drive superior sensitivity, sensing range, and signal clarity. A common metric used to evaluate resonant features is the quality factor *Q*, given by *Q* = λ_*m*_/Δλ_FWHM_.

The spring body optomechanical cavity is designed to detect changes in environmental pressure by deforming an enclosed cavity that contains air captured at atmospheric pressure. The cavity is sealed with optical adhesive after changing the position of the hinged mirror to retain the air inside the cavity. A positive pressure differential compresses the cavity, while a negative differential causes the cavity to expand. The spring body directs the deformation along the optical axis, so the hinged mirror maintains its original shape and curvature. The pressure exerted by the air inside the cavity is negligible, as the change in the cavity’s volume is small compared to the external pressure. To make a conservative estimate, we consider only the change in height predicted by eq 1. This analysis omits lateral deflection of the spring body and still indicates an internal pressure of only 0.25 ± 0.02%, the magnitude of the external pressure over the experimental range.

The mechanically adjustable top mirror serves the dual purpose of presenting the inner optical surface for thin-film deposition and sealing the cavity after the undeveloped resin is washed away. A flat lip is included around the top perimeter of the spring body to assist in leveling the hinged mirror. The natural stiction between the two flat surfaces also serves to align the mirror before placing the optical adhesive.

The spring body mimics a bellows or several Belleville washers in series. Each turn represents a spring element. More elements in series not only produce a lower spring stiffness which provides greater sensitivity and detection limits but also a longer cavity which leads to a smaller free spectral range (FSR) and heightens misalignment sensitivity. The spring body has a nominal thickness of 2 μm. The hinged mirror features a radius of curvature equal to 60 μm, and the length of the FP cavity is between 63.67 and 68.67 μm. This mechanically enabled optical cavity successfully demonstrated the utility of the spring body microchamber and the hinged mirror for encapsulating a working fluid.

**Figure 3:**
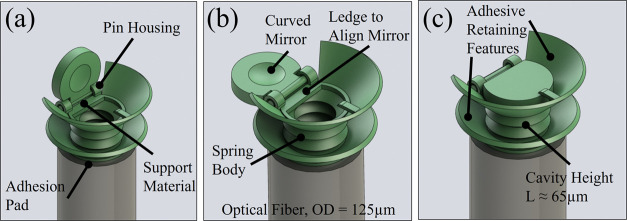
Graphics depicting the three positions of the spring body Fabry–Pérot cavity. (a) Device immediately after fabrication, before manipulating the mirror with a semiconductor analysis probe. (b) Device in the fully open position when the reflective film was deposited by passing the inner faces directly under the sputtering target. (c) Device in the closed position, ready to receive optical adhesive to seal the cavity.

Two conical adhesive-retaining features are included to direct the optical adhesive that seals the cavity of the spring body. The top feature also secures the hinged mirror in place. The lower feature prevents the base of the polymer structure from peeling away in response to the pressure differential. The stiction between the mirror and the lip of the spring body was sufficient to secure the mirror while the adhesive was applied. Figure 3 shows graphical illustrations of the spring body optomechanical cavity in several orientations to highlight its micromechanical features.

**Figure 4:**
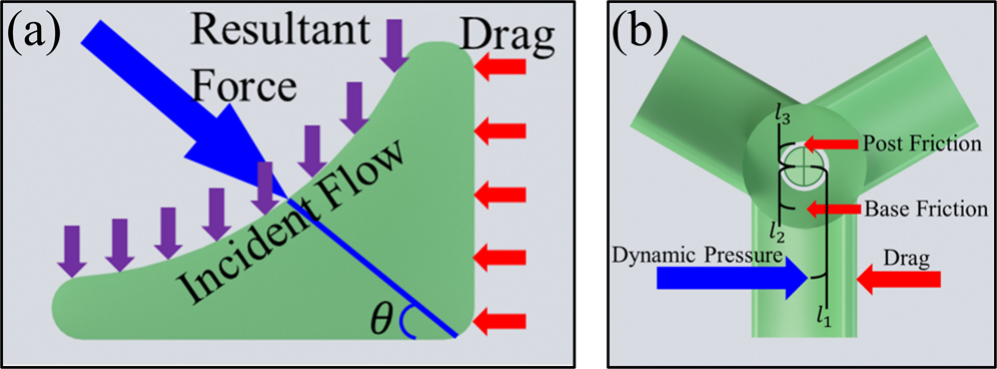
(a) Cross-sectional diagram depicting simplified aerodynamics at one rotor blade. (b) Top-view schematic depicting the simplified dynamic situation during operation.

In contrast to the resonant pressure sensor, the flow sensor relies on optical reflections to detect flows. Microblades are designed to rotate in response to incident flow; they spin more rapidly at higher flow rates. The emitted light from the fiber core is reflected back into the fiber by the planar surface on the bottom of the rotor blades as they pass over the fiber core. No light is reflected back into the fiber when the rotor blade is not over the fiber core. The flow regime used in this work is considered incompressible based on its Mach number, which was calculated to be 0.15 for the highest flow rate. This is below the common cutoff criterion of 0.3, which denotes the transition to a compressible regime.^61^ Following this assumption, the dynamic pressure exerted by the flow can be calculated using *P* = ρ*v*^2^/2, where ρ is the density of the flow and *v* is the velocity of the flow. The shape of the microblades causes both radial and axial reaction forces. The radial force drives the rotation, while the axial force presses the rotor into the base of the stator. Rotation is opposed by the drag of the blades, friction on the center post, and friction on the stator base, as illustrated in Figure 4a,b.

A sum of moments around the center of the stator can be approximated by
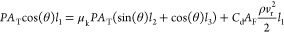
3where θ is the angle of the resultant force; μ_k_ is the coefficient of friction; *A*_T_ is the area of the top of the blade; *P* is the dynamic pressure; *l*_1_, *l*_2_, and *l*_3_ are the distances from the center of the stator to the center of the blade, the inner edge of the rotor, and the contact points on the base, respectively, as indicated in Figure 4b, *C*_d_ is the drag coefficient, *A*_F_ is the area of the front of the blades, and *v*_r_ is the rotational velocity of the blades.

## Experimental Section

The fiber tip microsystems were fabricated using two-photon polymerization (2PP) nanofabrication in a 2PP system, the Nanoscribe Photonic Professional GT. This technique is similar to stereolithography 3D printing, in which a liquid resin is selectively solidified by a laser that imparts the polymerization energy of the resin. This same polymerization energy can be achieved with two photons of light at half the frequency needed for single-photon polymerization. The volume within the laser beam capable of performing 2PP is much smaller than the volume capable of performing single-photon polymerization. This produces an extremely small voxel (3D pixel) that can be guided through the resin to solidify the desired volume. This work utilized a 200 nm × 200 nm × 200 nm voxel to fabricate the optical cavity with a monolithically integrated swivel mirror.

A Corning SMF-28e+ optical fiber was first cleaved using a Fujikura CT-30 high-precision fiber cleaver. It was then mounted into a Newport FPH-S fiber chuck, with a small portion (∼0.5 mm) extended out the end of the chuck. A drop of UV-curable resin, Nanoscribe’s IP-DIP, was then deposited onto the end of the fiber chuck, enveloping the fiber tip. The chuck was fixed to a custom 3D-printed jig, and the jig was fastened to the 2″ wafer plate provided with the Nanoscribe. This plate was used because it had three accessible threaded holes for mounting.

The laser aperture, a custom 63x objective lens, was then raised manually to contact the resin droplet on the fiber tip. The cleaved face of the fiber was located manually by the operator. A 5 μm thick pad was included as the base of the optical cavity to ensure adhesion to the fiber face. The starting height of the adhesion pad was selected manually by the operator to be below the surface of the fiber. This ensured polymerization began as close to the surface of the fiber as possible and secured the polymerized structure to the fiber.

**Figure 5:**
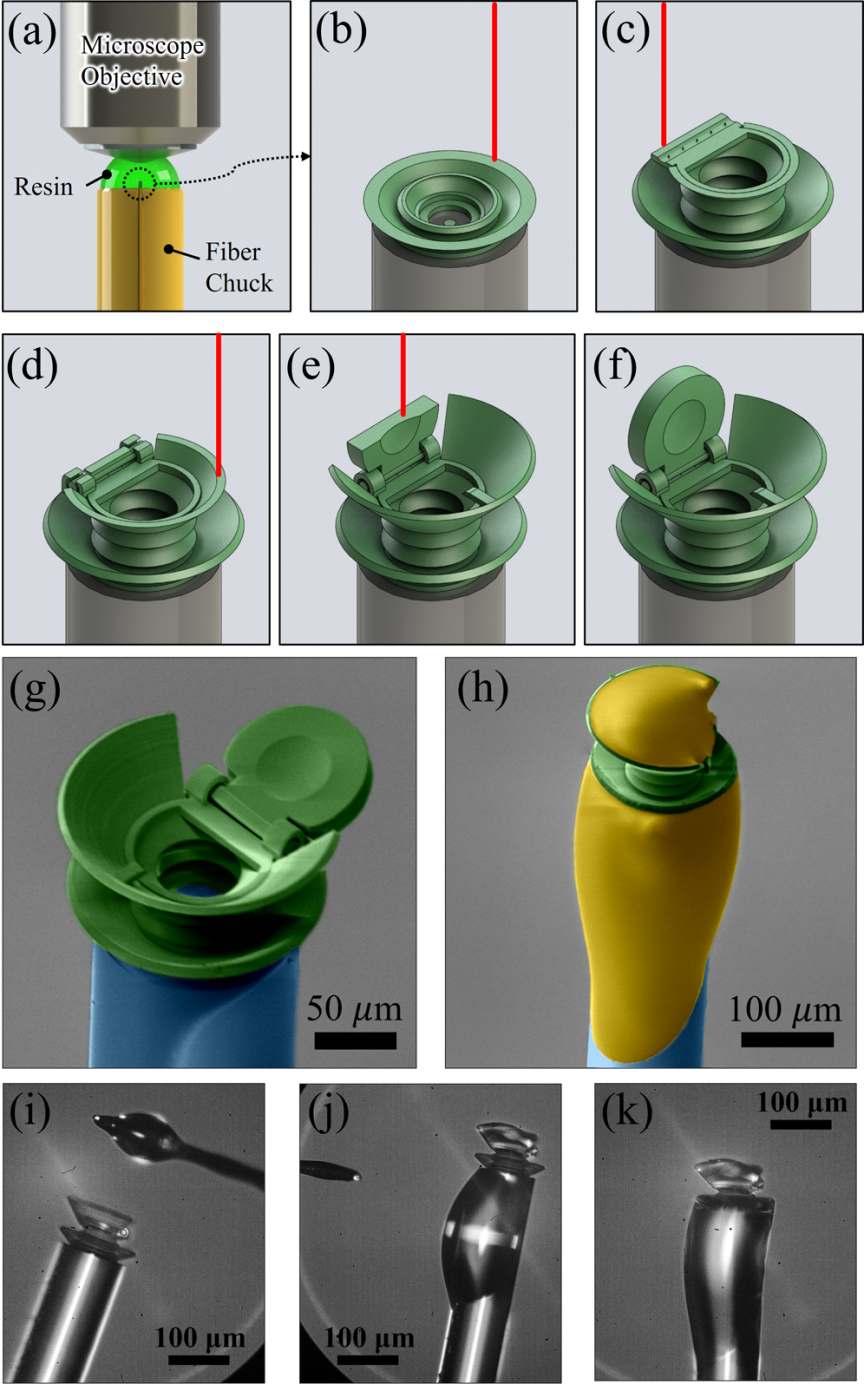
Graphical highlight of the 2PP fabrication process to create a mechanically assisted spring body FP cavity. (a) Microscope objective was immersed in a drop of resin on top of the fiber chuck surrounding the fiber tip. (b–e) Various stages of the two-photon polymerization process, as the device was constructed according to the sliced CAD file. (f) Final spring body FP cavity with a freely rotatable top mirror after the unexposed resin was washed away. (g) False-colored SEM image of the fabricated spring body FPC (green) in the half-open position on a fiber (blue) tip. (h) False-colored SEM image of the spring body FPC in the closed position after curing the optical adhesive (yellow). (i–k) Optical microscope images showing the UV-curable optical adhesive were applied to the upper and lower adhesive-retaining features of the spring body FPC.

Each device was designed using Solidworks 3D computer-aided design (CAD) software. The solid model was divided into thin layers by the DeScribe slicer software provided by Nanoscribe GmbH. Each layer was solidified by a mode-locked 780 nm laser with a 120 fs pulse duration, 80 MHz repetition rate, 40% laser power, and 10 mm/s scan speed directed by galvanometric control. Red light from a flashlight was coupled to the fiber to identify the core. A small disk on each device was used as an alignment mark to line up with the core, thus centering the device on the fiber tip. This was done by focusing the laser inside the fiber where it was still visible but not polymerizing resin. The laser inscription process flow of the mechanically assisted spring body FP cavity is highlighted in Figure 5a–f and animated in Video S1 (Supporting Information). The mechanical hinge allowed a curved mirror to be used in the cap to reduce misalignment sensitivity. Fabricating the spring body FPC in the open position also enabled the interior of the device to be easily cleared of undeveloped photoresin. A cavity could be made full of undeveloped resin, but exposure to sunlight or other UV sources would start to eventually polymerize this interior. The nonpolymerized resin was developed away in propylene glycol methyl ether acetate (PGMEA) for 20 min. Halfway through the development, the fiber was extended several millimeters for the remaining 10 min to ensure no droplets of resin formed around the device. The device was then cleaned in isopropyl alcohol (IPA) for 10 min to remove the PGMEA. Total fabrication time, including fiber preparation and mounting, multiphoton polymerization, resin development, and final device cleaning, was about 80 min.

Although 2PP nanofabrication is considerably faster than other nanofabrication techniques, the stepwise laser writing process presented striations into the surface finish of the fabricated structures. Hemispherical FP cavities require an optically smooth spherical mirror, and it was uncertain if the devices created here had an optical-quality surface finish. Also of concern, features with a height equal to one-half or one-quarter of the wavelength of interest could introduce destructive interference and create an antireflective surface. To analyze the surface finish, we fabricated a sample structure onto a glass slide to mount into a Bruker NanoScope V atomic force microscope (AFM). The resultant AFM scan is reported in Figure S2 (Supporting Information). The surface roughness from the stepwise laser writing process was present at regular intervals. The surface finish was found to have a roughness of less than 75 nm. This work focused on using wavelengths in the 1450–1650 nm range to probe the FP structures fabricated on the fiber ends. Therefore, the surface roughness is significantly smaller than the wavelengths of interest, and far less than one-half or one-quarter wavelength interval, which would lead to their own interference effects.

The research was performed to examine suitable highly reflective coating materials that can be deposited on the inner optical surfaces of the cavity to improve its reflectivity. Glass slides (76 mm × 24 mm × 1 mm) were coated on each side with thin-film dielectrics and metals. A series of measurements to select a coating material that yields the highest optical *Q*-factor were performed according to the experimental setup in Figure S3a (Supporting Information). Results plotted in Figure S3b (Supporting Information) suggest that 20 nm of gold deposited by a Kurt J. Lesker Company LAB 18 magnetron plasma sputtering system should be employed as a reflective coating material to enhance the *Q*-factor of the cavity.

To deposit a thin film of gold, the top rotatable mirror was moved into the fully open position using a semiconductor analysis probe. Manually manipulating the top rotatable mirror required dexterity similar to precise wire-bonding or device probing. A thin layer of gold was then deposited onto the interior of the hinged mirror and the fiber face by a magnetron sputtering system. The sputtering parameters for the device are listed in Table S1 (Supporting Information). The hinged mirror was manipulated to the fully open position, such that the inner-curved surface was parallel to the fiber face and facing away from the fiber surface, as illustrated in Figure 1a. Both the curved surface and fiber face were oriented toward the sputtering target by holding the fiber in a custom spring-loaded jig. The spring body FPC was positioned in the center of the chamber on a rotating platen, with the target facing the platen at an acute angle. The hinged mirror was then lowered into its final position, again using a semiconductor analysis probe. The flexibility of the solidified polymer allowed moderately aggressive manipulations without breaking the device. The spring body FPC was then sealed with Norland optical adhesive 68 (NOA 68). The adhesive was beaded onto a wire-style probe, then applied to the top adhesive-retaining feature. This droplet was cured using a CS2010 UV curing system from Thorlabs, Inc. for 3 min. The second bead of adhesive was then applied to the lower adhesive-retaining feature and cured for 4 min. This sealed the cavity at atmospheric pressure. The false-colored scanning electron microscope (SEM) images of the fabricated mechanically assisted spring body FPC in open and closed positions are shown in Figure 5g,h. The optical microscope images showing the UV-curable optical adhesive was applied to the upper and lower adhesive-retaining features of the spring body FPC are presented in Figure 5i–k.

**Figure 6:**
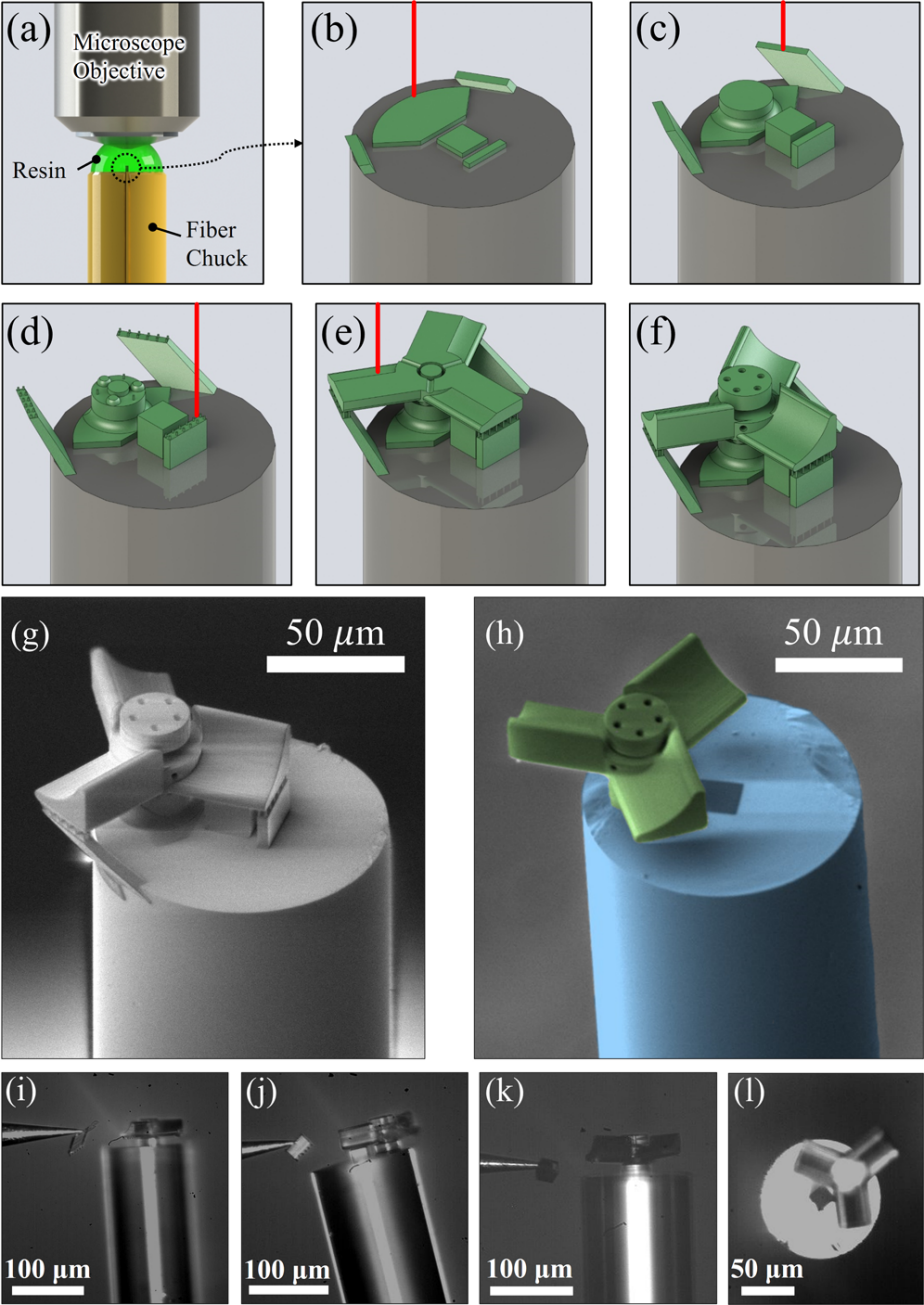
Graphical highlight of the 2PP fabrication process to create a fiber tip microturbine flow sensor. (a) Microscope objective was immersed in a drop of resin on top of the fiber chuck surrounding the fiber tip. (b–e) Various stages of the two-photon polymerization process, as the device was constructed according to the sliced CAD file. (f) Final microturbine flow sensor with breakable support structures for each rotor blade and a masking cap over the core of the fiber after the unexposed resin was washed away. (g) SEM image of the flow sensor before removal of the support structures and masking cap. (h) False-colored SEM image of the final device after removal of all support structures and masking cap. (i, j) Optical microscope images showing the probe removing support structures. (k) Optical microscope image showing the probe removing the masking cube. (l) Top-view optical microscope image of the released microturbine.

The optical nonresonant microturbine flow sensor was also fabricated using the same parameters of the 2PP process. The laser inscription process of the microturbine flow sensor is highlighted in Figure 6a–f and animated in Video S2 (Supporting Information). The device was fabricated with breakable support structures for each rotor blade and a masking cap over the core of the fiber. Twelve 1 μm pillars connected the blades to the support structures. The masking cap was a 20 μm cube. The inner pillar of the stator has a diameter 12 μm and a clearance of 2 μm with the rotor. Holes were patterned to encourage the PGMEA solution to enter these tight clearances. Three hemispherical features were patterned on the base of the stator to reduce friction with the rotor.

The SEM image of the optical nonresonant microturbine flow sensor with breakable support structures and a masking cap to protect the core of the fiber during metal deposition is displayed in Figure 6g. The false-colored SEM image of the ready-to-operate device after the removal of support structures and masking cap is shown in Figure 6h. The support material was removed by mounting the fiber into a Newport FPH-S side loading fiber chuck, which was loaded into a Newport 561-FC fiber chuck holder. This stainless-steel block was placed under a Micromanipulator probe station. A Jmicro Technology KRN- 09S magnetic probe arm was used with a Pacific Instruments ST-1 100 nm diameter semiconductor analysis probe to remove the support material, as pictured in Figure 6i,j. After the support structures were removed, a thin layer of gold was then deposited onto the top surface of the microblades by a Kurt J. Lesker Company LAB 18 magnetron plasma sputtering system. The fiber was oriented perpendicular to the sputtering target, placing the bottoms of the blades at a 90° angle. The sputtering parameters for the device are listed in Table S2 (Supporting Information). The masking cube was then removed with the probe, as shown in Figure 6k, revealing the core of the fiber (Figure 6l). This was best accomplished by pushing high on the masking cube with the very tip of the probe to peel it off of the fiber. After this step, the microturbine flow sensor was ready to test.

## Results and Discussion

**Figure 7:**
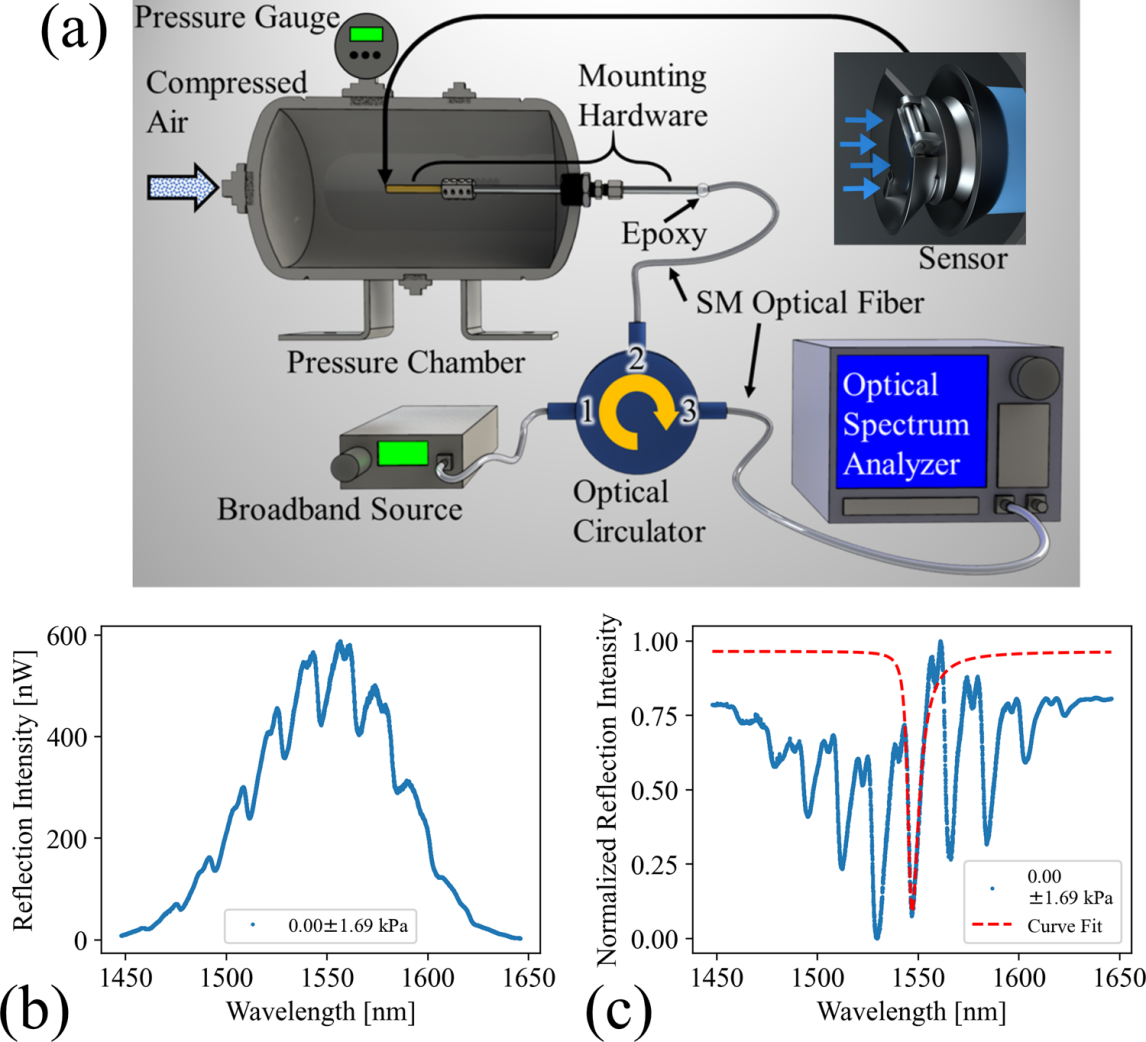
(a) Schematic describing the experimental setup used to characterize the reflection intensity of the fabricated spring body FPC on an optical fiber tip. (b) Preprocessed reflection spectrum of the spring body FPC. (c) Same reflection spectrum after subtracting the baseline spectral power dependence profile of the SLD and normalizing between minimum and maximum reflection values. The red dashed line shows the result of fitting eq 4 to a chosen resonance feature.

The spring body FPC was characterized according to the experimental setup described in Figure 7a. A 6015–3 optical circulator from Thorlabs, Inc. was used to isolate the reflection spectrum out of the spring body FPC. The optical circulator used here was a standard fiber-optic device that transmitted light from ports 1 to 2 and 2 to 3 (with ∼1 dB of insertion loss) and prevented transmission in the opposite direction (∼40 dB of attenuation). A fiber-coupled superluminescent diode (SLD) broadband source (BBS), the Thorlabs S5FC1550SP-A2, was connected to port 1, which emitted a 200 nm spectrum centered at 1550 nm. The intensity of the SLD was wavelength-dependent, but the profile of this dependence can be easily subtracted from the device measurements. The fabricated spring body FPC was connected to port 2 of the optical circulator. The reflection spectrum from the device was isolated and routed through port 3 to a Yokogawa AQ6370C optical spectrum analyzer (OSA).

The baseline wavelength power dependence of the BBS was recorded from the average of five measurements of the BBS routed directly into the OSA, removing the effects of the optical circulator. This baseline was subtracted from each measured reflection spectrum of the devices. The magnitude of the response was then normalized to the minimum and maximum reflection intensity of that reading. Measurements plotted before and after this processing are presented in Figure 7b,c. The ideal output of the FP resonator is the Airy function, which can be analyzed as the sum of individual resonant features with Lorentzian profiles.^59^ Individual resonant features were analyzed in this work to evaluate the shift produced by the environmental stimuli with higher fidelity than analyzing the entire spectral response. The peak wavelength and FWHM of the resonant features were approximated by fitting a generic skew-Lorentzian distribution to 500 points around each feature. The generic skew-Lorentzian expression^62^ used for the fitting is
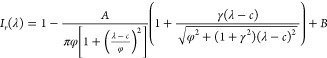
4Best-fit values were found for *A*, a scaling factor, *B*, an offset factor, *c*, the center wavelength of a nonskewed curve, φ, the FWHM of a nonskewed curve, and γ, the skew factor. Both *c* and φ lost their physical meaning on a skewed curve, so the peak and FWHM were calculated from the output of the fit using the BBS wavelength spectrum from the experiment as the input. The curve was subtracted from 1, which represented full transmission, to properly orient the feature as a dip rather than a peak. The curve generated by fitting eq 4 to the measured reflection data is presented in Figure 7c.

Equation 4 was chosen for the functional model because it can capture the asymmetry observed around the resonant features and more accurately identified the minimum of the spectral response, which was used to indicate changes in the cavity. The asymmetrical response has been observed in prior literature and was attributed to a phase shift introduced by a high reflective metal coating near the limited aperture in a single-mode fiber.^63^ This skewed shape was also present in multimode fiber FP resonators and was attributed to multiple resonant modes propagating within the cavity near the ideal resonant wavelength.^64^

To measure environmental pressure, the fiber tip spring body FPC was enclosed in a pressure chamber, as shown in Figure 7. The device fiber was mounted in a side-loading fiber chuck, which was connected by a shaft coupler to a stainless-steel tube. A compression fitting compatible with the threading of the pressure chamber was affixed to the tube to create a seal. The device fiber was fed through the tube, and the open end was sealed with epoxy resin. This mounting assembly was then threaded into the pressure chamber. A vacuum pump or compressor was connected to an open port in the chamber to measure vacuum and positive pressure, respectively. A Weiss Solarmetrix pressure gauge was used to monitor the pressure inside the chamber, measuring vacuum first. The setup was held static for approximately 60 s when a measurement pressure was reached. The reflection spectrum was then measured three times. When atmospheric pressure was reached, the vacuum pump was removed, the compressor was attached, and positive pressure measurements were taken in the same manner. The total pressure testing range was −80 to 345 kPa.

**Figure 8:**
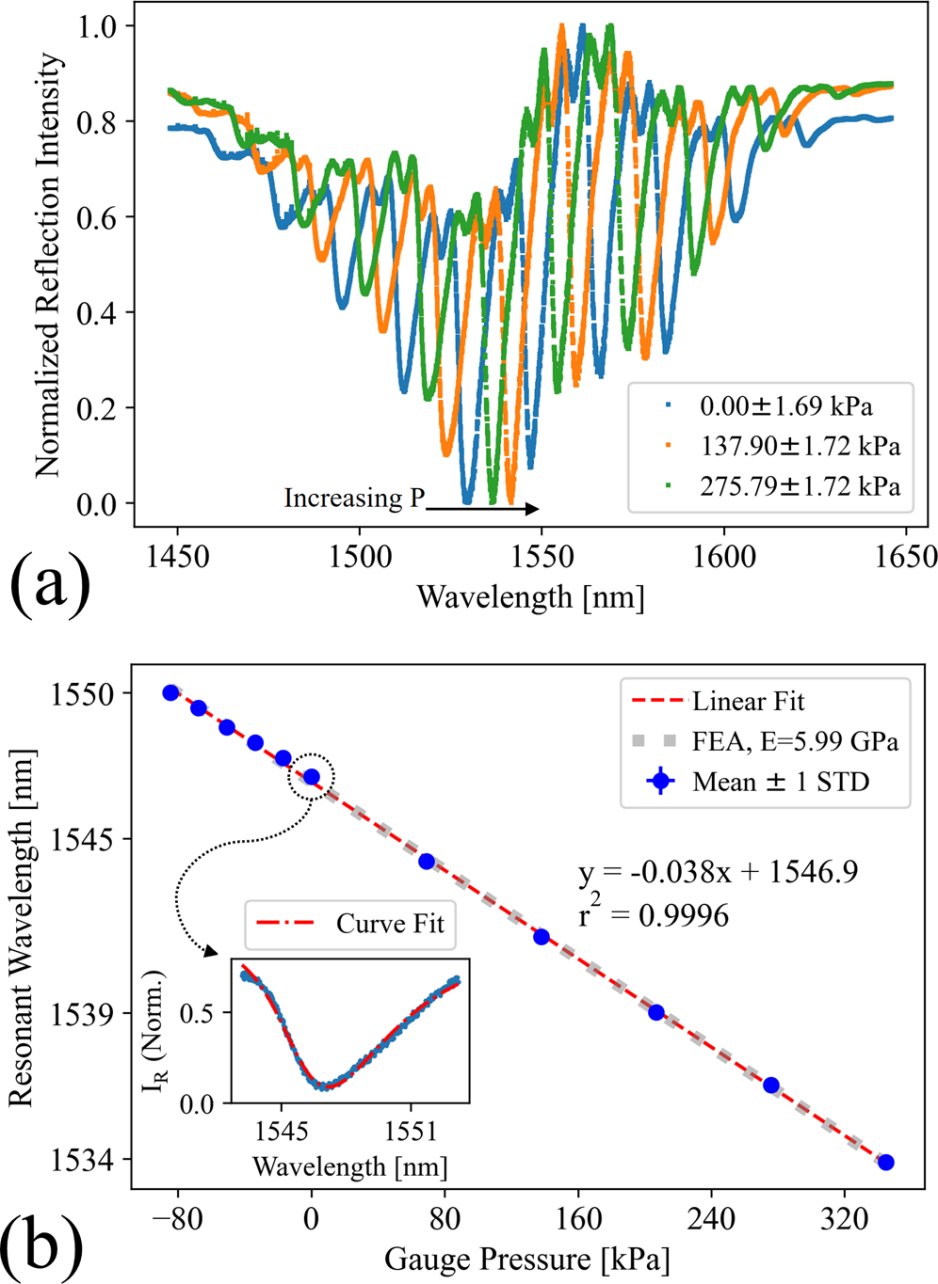
Results of the pressure-sensing experiment with the spring body FP pressure sensor. (a) Three reflection spectra from the device at different pressures. Each response has been normalized between its minimum and maximum reflection values and had the SLD’s baseline spectral power dependence profile subtracted. The primary resonant features are noted with an arrow showing increasing pressure, while smaller, off-axis resonant features can be seen between the primary features. (b) Locations of peak wavelengths of the primary resonant features as extracted from fitting a skewed Lorentzian distribution at their respective pressures. The points represent the mean of three measurements taken at a given pressure, the vertical error bars represent one standard deviation of these measurements, and the horizontal error bars represent the reported accuracy of the reference pressure gauge. The red, long dashed line represents the linear fit to this data. The gray, small, dashed line is the resonant wavelength shift predicted by eq 1 with a modulus of elasticity extracted from finite element analysis (FEA). The inset shows the curve fitting on top of the measured reflection response at atmospheric pressure.

The spring body FPC exhibited a linear shift in resonant wavelength in response to environmental pressure, as shown in Figure 8. The observed blueshift at positive pressures and redshift at vacuum was consistent with compression and expansion of the spring body, respectively, as predicted by eq 1. A sensitivity of 38 ± 7 pm/kPa was recorded across the combination of vacuum and positive pressures. The uncertainty listed represents the 95% confidence interval of the slope in the linear fit. The results of pressure sensing from −80 to 345 kPa are presented in Figure 8b. The blue points represent the means of the three measurements taken at each pressure. The vertical error bars show one standard deviation of these measurements, while the horizontal error bars show the reported accuracy of the commercial pressure gauge. The sensitivity of the fiber tip spring body FPC in this work is comparable or better than some of the previously demonstrated fiber tip pressure sensors.^2,4,16^

The fitted curves yielded an average *Q*-factor of 224 ± 12 and mirror reflectance of 0.3081 ± 0.0211. The quality factor of this device would benefit from both securing the mirror and shortening the cavity, although fewer spring elements in series will reduce sensitivity. A thorough optimization could be performed to find an optimal balance of these properties. One secondary resonant feature, again caused by an off-axis mode, can be observed in Figure 8a between the primary features. Other off-axis resonant features, and indeed parts of the primary feature, are obscured by the constructive interference between resonant events with a larger FWHM and smaller FSR.

**Figure 9:**
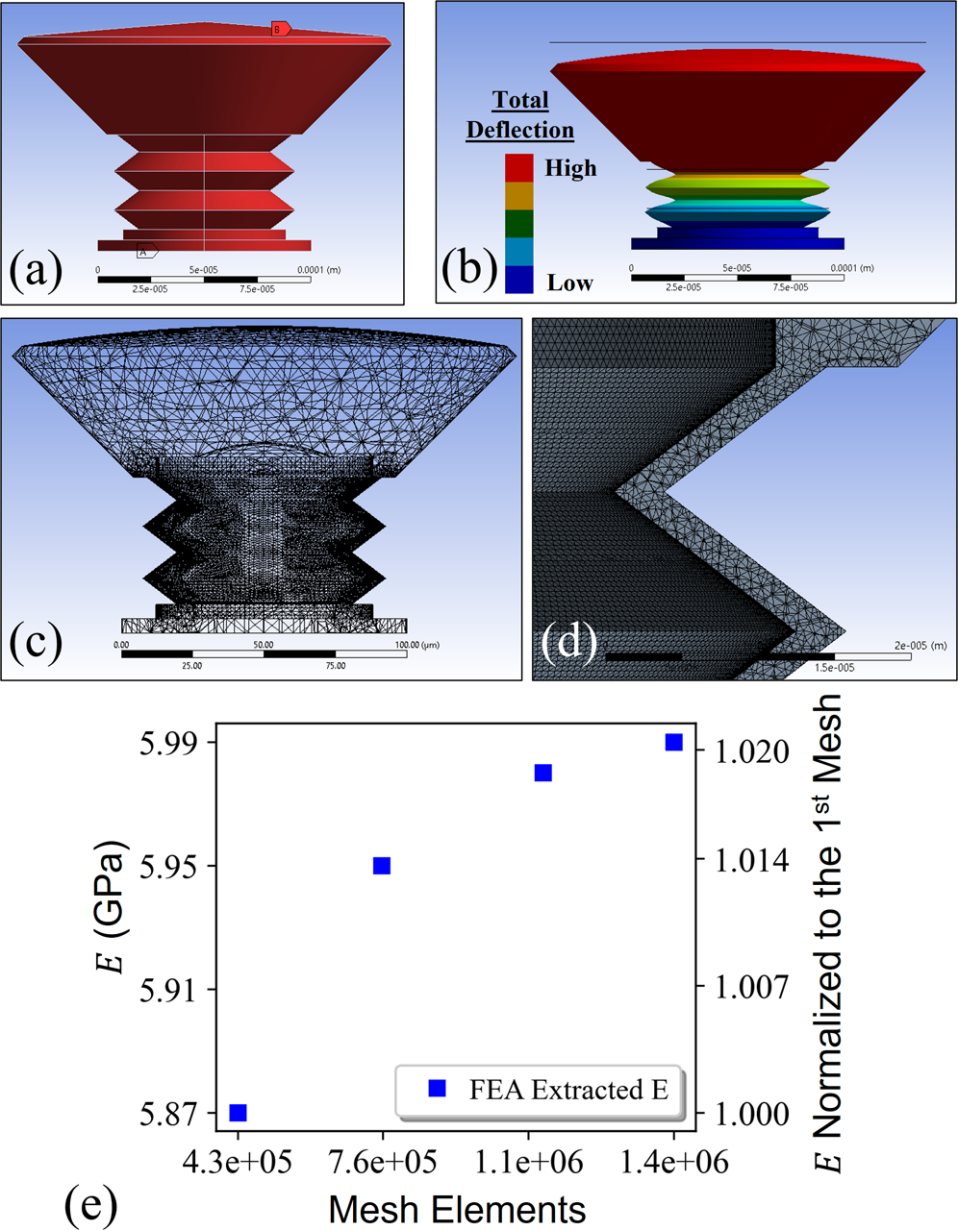
FEA model used to extract a plausible modulus of elasticity for the spring body device. (a) Representative pressure was applied to all external surfaces (denoted by B) and the bottom surface in contact with the fiber was fixed (denoted by A). (b) Exaggerated depiction of the deflection predicted by the model. (c) View of the mesh used in the model, which was greatly refined around the spring body elements. (d) Magnified view of the mesh used in the spring body elements. (e) Results of a mesh refinement study.

Equation 1 was used to approximate the change in cavity length that produced the observed shift in resonant wavelength. The exact height of the cavity can range between 63.67 and 68.67 μm because the fabrication surface was manually located, so an approximated cavity height of 65 μm was used. A simplified model of the spring body device was investigated using finite element analysis (FEA) to extract a plausible modulus of elasticity for the cured resin. The Poisson’s ratio, yield strength, and density of the material were chosen to be 0.49, 70 MPa, and 1250 kg/m^3^ after reviewing common values found in literature for the IP-Dip resin.^65−68^ The modulus of elasticity was then varied until the FEA model predicted the same displacement as eq 1, within three significant figures, for a given pressure applied to all external surfaces of the device. A mesh refinement study was performed to raise confidence in the extracted value. This process resulted in a modulus of elasticity of approximately 6 GPa. This result is on the high end of reported literature values but aligns well with reports of resin intentionally stiffened by UV curing.^65^ The UV cure for the optical adhesive likely caused a similar stiffening in our device. The 3D model, the modeled deflection mode, a portion of the mesh, and the results of the mesh refinement study are presented in Figure 9.

**Figure 10:**
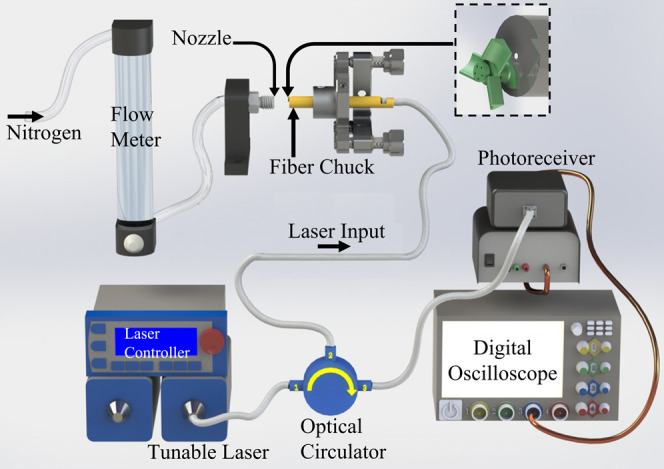
Schematic describing the experimental setup used to characterize the reflection intensity of the microturbine flow sensor.

The microturbine flow sensor was characterized according to the experimental setup described in Figure 10. The reflected light from the microblades was isolated with a 6015–3 optical circulator from Thorlabs, Inc. A Sacher LaserTechnik TEC 520 1550 nm laser was connected to the first port, the microturbine flow sensor was connected to the second port, and a Newport model 1611 1 GHz low-noise photoreceiver was connected to the third port of the optical circulator. The fiber tip flow sensor was mounted into a fiber chuck and a mirror mount, monitored with an optical microscope and oriented to maximize rotational velocity and consistency.

Compressed nitrogen was connected to an analog flow controller. A 1/4″ to 1/8″ compression fitting reducer was used as the outlet nozzle. Two flow ranges were evaluated, a coarse range from 9 to 25 LPM with steps of 1.7 LPM and a fine range from 10.9 to 12 LPM with steps of 0.17 LPM. These values utilized the full operating range and smallest graduations of our flow meter, respectively. The fine range was chosen around the greatest rate of change observed over the coarse range. While spinning, the reflection spectrum of the device was recorded over 0.5 s with an Agilent 54641D mixed-signal oscilloscope. Five measurements were taken at each flow rate. A movie that shows a rotating flow sensor in response to incident nitrogen flow can be seen in Video S3 (Supporting Information).

**Figure 11:**
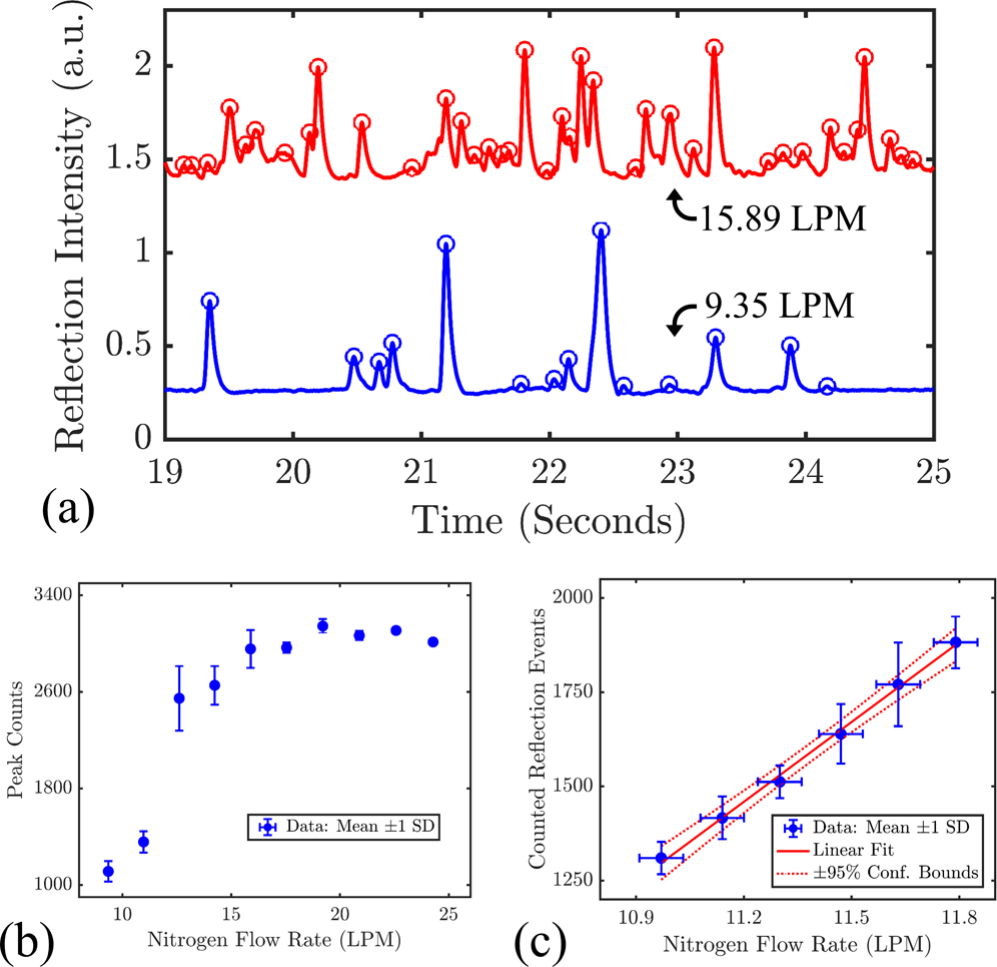
(a) Sample reflection spectra from the microturbine flow sensor at 9.35 and 15.89 LPM of nitrogen flow rate. (b) Reflection peak counts of the fiber tip flow sensor from 9 to 25 LPM of nitrogen flow rate. (c) Reflection response of the fiber tip flow sensor in a linear regime. Vertical error bars represent one standard deviation from the mean of repeated measurements. The linear fit and 95% confidence bounds are included as the solid and dashed lines, respectively.

The device’s rotational velocity is directly correlated with the flow rate incident on the microblades; however, the rotation of the microblades was erratic and did not produce a steady-state rotation. Nevertheless, the relationship can be quantified by summing the reflection events over the 0.5 s measurement. A lowpass, moving average filter was applied to the reflection response of each measurement. Reflection peaks, caused by a rotor blade passing over the fiber core, were counted using a peak-finding function. The same process was applied to each measurement. Two examples of this reflection counting for a nitrogen flow rate of 9.35 and 15.89 LPM are presented in Figure 11a.

We hypothesize that the rotational irregularity originated from three factors: dry, sticky friction between the rotor and stator, backflow from the fiber face, and inconsistent contact caused by the rotor clearances. Friction and contact could be improved by implementing integrated bearings, adding lubricant, or operating in a liquid. Backflow may be reduced by measuring flow parallel to the fiber face, although this would increase shearing stresses on the base.

The flow sensor functioned over a wide flow range but exhibited nonlinear behavior at this scale, as reported in Figure 11b. Greater backflow from the fiber face likely slowed down the rotor at these higher flow rates. Within the smaller flow range, the reflection response is effectively a linear approximation of the larger trend, as displayed in Figure 11c. The vertical error bars represent one standard deviation of the mean from repeated measurements, the horizontal error bars represent the repeatability reported for the flow meter, and the red lines represent a linear fit with the 95% confidence band. This smaller range is more appropriate for precise sensing since the response is primarily linear over the selected flow rates. A sensitivity of 706 ± 43 reflections/LPM, including one coefficient standard error, was observed from the linear fit. The demonstrated that microturbine flow sensor in this work offers a new nonresonant flow sensing mechanism that will be complementary to the previously demonstrated resonant-based flow sensing techniques.^39,42^

## Conclusions

This work demonstrates a breakthrough combination of design and nanostructuring process for micro-optomechanical systems: a spring body FPC with dynamically rotatable mirror and a microturbine flow sensor fabricated with submicron accuracy by multiphoton nanosculpting method to enable integration and fabrication techniques beyond the limits of the current technology. This capability facilitates the creation of improved SWAP micro-optomechanical systems for precise multipurpose sensing. Microscale fiber tip optical resonant and nonresonant sensors with advanced micromechanical features and an enhanced internal cavity were designed and fabricated. The fabricated resonant and nonresonant sensors successfully demonstrated sensing of pressure and flow. Sensitivities of 38 ± 7 pm/kPa for pressure sensing and 706 ± 43 reflections/LPM for flow sensing were achieved. We currently explore novel locking and self-aligning mechanisms to stabilize the integrated components created by the multiphoton nanosculpting technique. In addition, we also investigate advanced coating options to improve the *Q*-factors of future resonant devices. The enhancement of *Q*-factors by 1–2 orders of magnitudes will open doors to broader sensing and signal processing applications and support other fundamental scientific endeavors. The mechanically enhanced 3D optical microsystems demonstrated in this work present a powerful enabling technology for meeting a variety of difficult integration and fabrication challenges that are currently limiting the research progress in microscale optics and other related fields.

## Supporting Information Available

Measured reflection intensity before and after reflective coating deposition, surface analysis of an optical flat fabricated with two-photon polymerization by ultrashort laser pulses, experimental study to select a coating material that yields the highest reflectivity, a video animation of the laser inscription process to create a spring body Fabry–Pérot cavity, a video animation of the laser inscription process to create a microturbine flow sensor, a movie that shows a rotating flow sensor in response to incident nitrogen flow, sputtering settings used to deposit the gold reflective coating for the spring body FPC, and sputtering settings used to deposit the gold reflective coating for the microturbine flow sensor (PDF)
Video S1 (MP4)
Video S2 (MP4)
Video S3 (MP4)

## References

[ref1] Wu J.; Yao M.; Xiong F.; Zhang A. P.; Tam H.-Y.; Wai P. K. A. Optical Fiber-Tip Fabry–Pérot Interferometric Pressure Sensor Based on an In Situ μ-Printed Air Cavity. J. Lightwave Technol. 2018, 36, 3618–3623. 10.1109/JLT.2018.2843885.

[ref2] Yao M.; Ouyang X.; Wu J.; Zhang A. P.; Tam H.-Y.; Wai P. K. A. Optical Fiber-Tip Sensors Based on In-Situ μ-Printed Polymer Suspended-Microbeams. Sensors 2018, 18, 1825 10.3390/s18061825. PMC6022165 29874800

[ref3] Liu J.; Jia P.; Zhang H.; Tian X.; Liang H.; Hong Y.; Liang T.; Liu W.; Xiong J. Fiber-Optic Fabry–Perot Pressure Sensor Based on Low-Temperature Co-Fired Ceramic Technology for High-Temperature Applications. Appl. Opt. 2018, 57, 4211–4215. 10.1364/AO.57.004211. 29791395

[ref4] Tan X.; Li X.; Geng Y.; Yin Z.; Wang L.; Wang W.; Deng Y. Polymer Microbubble-Based Fabry–Perot Fiber Interferometer and Sensing Applications. IEEE Photonics Technol. Lett. 2015, 27, 2035–2038. 10.1109/LPT.2015.2449654.

[ref5] Quan M.; Tian J.; Yao Y. Ultra-High Sensitivity Fabry–Perot Interferometer Gas Refractive Index Fiber Sensor Based on Photonic Crystal Fiber and Vernier Effect. Opt. Lett. 2015, 40, 4891–4894. 10.1364/OL.40.004891. 26512476

[ref6] Ma W.; Jiang Y.; Hu J.; Jiang L.; Zhang T.; Zhang T. Microelectromechanical System-Based, High-Finesse, Optical Fiber Fabry–Perot Interferometric Pressure Sensors. Sens. Actuators, A 2020, 302, 111795 10.1016/j.sna.2019.111795.

[ref7] Yang X.; Wu S.; Cheng H.; Ma J.; Wang S.; Liu S.; Lu P. Simplified Highly-Sensitive Gas Pressure Sensor Based on Harmonic Vernier Effect. Opt. Laser Technol. 2021, 140, 107007 10.1016/j.optlastec.2021.107007.

[ref8] Hill G. C.; Melamud R.; Declercq F. E.; Davenport A. A.; Chan I. H.; Hartwell P. G.; Pruitt B. L. SU-8 MEMS Fabry-Perot Pressure Sensor. Sens. Actuators, A 2007, 138, 52–62. 10.1016/j.sna.2007.04.047.

[ref9] Wei H.; Chen M.; Krishnaswamy S. Three-Dimensional-Printed Fabry–Perot Interferometer on an Optical Fiber Tip for a Gas Pressure Sensor. Appl. Opt. 2020, 59, 2173–2178. 10.1364/AO.385573. 32225743

[ref10] Coote J. M.; Alles E. J.; Noimark S.; Mosse C. A.; Little C. D.; Loder C. D.; David A. L.; Rakhit R. D.; Finlay M. C.; Desjardins A. E. Dynamic Physiological Temperature and Pressure Sensing with Phase-Resolved Low-Coherence Interferometry. Opt. Express 2019, 27, 5641–5654. 10.1364/OE.27.005641. 30876162 PMC6410922

[ref11] Bae H.; Yun D.; Liu H.; Olson D. A.; Yu M. Hybrid Miniature Fabry–Perot Sensor with Dual Optical Cavities for Simultaneous Pressure and Temperature Measurements. J. Lightwave Technol. 2014, 32, 1585–1593. 10.1109/JLT.2014.2308060.

[ref12] Guo X.; Zhou J.; Du C.; Wang X. Highly Sensitive Miniature All-Silica Fiber Tip Fabry–Perot Pressure Sensor. IEEE Photonics Technol. Lett. 2019, 31, 689–692. 10.1109/LPT.2019.2904420.

[ref13] Pevec S.; Donlagic D. Miniature All-Fiber Fabry–Perot Sensor for Simultaneous Measurement of Pressure and Temperature. Appl. Opt. 2012, 51, 4536–4541. 10.1364/AO.51.004536. 22772127

[ref14] Guo F.; Fink T.; Han M.; Koester L.; Turner J.; Huang J. High-Sensitivity, High-Frequency Extrinsic Fabry–Perot Interferometric Fiber-Tip Sensor Based on a Thin Silver Diaphragm. Opt. Lett. 2012, 37, 1505–1507. 10.1364/OL.37.001505. 22555719

[ref15] Cheng X.; Dash J. N.; Gunawardena D. S.; Htein L.; Tam H.-Y. Silicone Rubber Based Highly Sensitive Fiber-Optic Fabry–Perot Interferometric Gas Pressure Sensor. Sensors 2020, 20, 4927 10.3390/s20174927. PMC7506645 32878217

[ref16] Zhang Z.; Liao C.; Tang J.; Bai Z.; Guo K.; Hou M.; He J.; Wang Y.; Liu S.; Zhang F.; Wang Y. High-Sensitivity Gas-Pressure Sensor Based on Fiber-Tip PVC Diaphragm Fabry–Pérot Interferometer. J. Lightwave Technol. 2017, 35, 4067–4071. 10.1109/JLT.2017.2710210.

[ref17] Poduval R. K.; Coote J. M.; Mosse C. A.; Finlay M. C.; Desjardins A. E.; Papakonstantinou I. Precision-Microfabricated Fiber-Optic Probe for Intravascular Pressure and Temperature Sensing. IEEE J. Sel. Top. Quantum Electron. 2021, 27, 1–12. 10.1109/JSTQE.2021.3054727. PMC7951063 33716587

[ref18] Liu Y.; Jing Z.; Li R.; Zhang Y.; Liu Q.; Li A.; Zhang C.; Peng W. Miniature Fiber-Optic Tip Pressure Sensor Assembled by Hydroxide Catalysis Bonding Technology. Opt. Express 2020, 28, 948–958. 10.1364/OE.380589. 32121814

[ref19] Zhang W.; Li H.; Zhu L.; Dong M.; Meng F. Dual-Parameter Optical Fiber Probe Based on a Three-Beam Fabry-Perot Interferometer. IEEE Sens. J. 2021, 21, 4635–4643. 10.1109/JSEN.2020.3034915.

[ref20] Li X.; Warren-Smith S. C.; Xie L.; Ebendorff-Heidepriem H.; Nguyen L. V. Temperature-Compensated Refractive Index Measurement Using a Dual Fabry–Perot Interferometer Based on C-Fiber Cavity. IEEE Sens. J. 2020, 20, 6408–6413. 10.1109/JSEN.2020.2976626.

[ref21] Smith J. W.; Williams J. C.; Suelzer J. S.; Usechak N. G.; Chandrahalim H. Three-Dimensional Fabry–Pérot Cavities Sculpted on Fiber Tips Using a Multiphoton Polymerization Process. J. Micromech. Microeng. 2020, 30, 125007 10.1088/1361-6439/abc0fd.

[ref22] Smith J. W.; Suelzer J. S.; Usechak N. G.; Tondiglia V. P.; Chandrahalim H. In 3-D Thermal Radiation Sensors on Optical Fiber Tips Fabricated Using Ultrashort Laser Pulses, 20th International Conference on Solid-State Sensors, Actuators and Microsystems Eurosensors XXXIII (Transducers Eurosensors XXXIII) 2019; pp 649–652.

[ref23] Mathew J.; Hauser C.; Stoll P.; Kenel C.; Polyzos D.; Havermann D.; MacPherson W. N.; Hand D. P.; Leinenbach C.; Spierings A.; Koenig-Urban K.; Maier R. R. J. Integrating Fiber Fabry-Perot Cavity Sensor Into 3-D Printed Metal Components for Extreme High-Temperature Monitoring Applications. IEEE Sens. J. 2017, 17, 4107–4114. 10.1109/JSEN.2017.2703085.

[ref24] Li M.; Liu Y.; Gao R.; Li Y.; Zhao X.; Qu S. Ultracompact Fiber Sensor Tip Based on Liquid Polymer-Filled Fabry-Perot Cavity with High Temperature Sensitivity. Sens. Actuators, B 2016, 233, 496–501. 10.1016/j.snb.2016.04.121.

[ref25] Yang K.; He J.; Wang Y.; Liu S.; Liao C.; Li Z.; Yin G.; Sun B.; Wang Y. Ultrasensitive Temperature Sensor Based on a Fiber Fabry–Pérot Interferometer Created in a Mercury-Filled Silica Tube. IEEE Photon. J. 2015, 7, 1–9. 10.1109/JPHOT.2015.2504960.

[ref26] Liu G.; Han M.; Hou W. High-Resolution and Fast-Response Fiber-Optic Temperature Sensor Using Silicon Fabry-Pérot Cavity. Opt. Express 2015, 23, 7237–7247. 10.1364/OE.23.007237. 25837068

[ref27] Kou J.; Feng J.; Ye L.; Xu F.; Lu Y. Miniaturized Fiber Taper Reflective Interferometer for High Temperature Measurement. Opt. Express 2010, 18, 14245–14250. 10.1364/OE.18.014245. 20588559

[ref28] Chandrahalim H.; Smith J. Temperature-Immune Self-Referencing Fabry-Pérot Cavity Sensors. U.S. Patent US10942313B2, March 9, 2021.

[ref29] Smith J. W.; Williams J. C.; Suelzer S. J.; Usechak N. G.; Chandrahalim H. 3-D Optical Cavities Created Using Local Light-Triggered Polymerization on Fiber Tips. In Conference on Lasers and Electro-Optics (2020), paper ATu3K.6; Optical Society of America, 2020; Vol. 56, https://doi.org/10.1364/CLEO_AT.2020.ATu3K.6.

[ref30] Chandrahalim H.; Williams J. C.; Smith J. W.; Suelzer J. S.; Usechak N. G. In Micromechanically Enabled Microcavity on Optical Fiber Tips, IEEE Research and Applications of Photonics in Defense Conference (RAPID), 2021; Vol. 1–2.

[ref31] Chandrahalim H.; Smith J. Method of Making Temperature-Immune Self-Referencing Fabry-Pérot Cavity Sensors. U.S. Patent US11156782B2, October 26, 2021.

[ref32] Williams J.; Chandrahalim H. Hinged Temperature-Immune Self-Referencing Fabry-Pérot Cavity Sensors. U.S. Patent US20210271027A1, September 2, 2021.

[ref33] Williams J.; Chandrahalim H. Method of Making Hinged Self-Referencing Fabry-Pérot Cavity Sensors. U.S. Patent US11287575B2, March 29, 2022.

[ref34] Williams J.; Smith J.; Suelzer J. S.; Usechak N. G.; Chandrahalim H. Optical Fiber-Tip Heat Sensor Featuring a Multipositional Fabry–Pérot Cavity Resonator. 2020 IEEE Sensors 2020, 1–4. 10.1109/SENSORS47125.2020.9278730.

[ref35] Liao C. R.; Hu T. Y.; Wang D. N. Optical Fiber Fabry-Perot Interferometer Cavity Fabricated by Femtosecond Laser Micromachining and Fusion Splicing for Refractive Index Sensing. Opt. Express 2012, 20, 22813–22818. 10.1364/OE.20.022813. 23037431

[ref36] Wei T.; Han Y.; Li Y.; Tsai H.-L.; Xiao H. Temperature-Insensitive Miniaturized Fiber Inline Fabry-Perot Interferometer for Highly Sensitive Refractive Index Measurement. Opt. Express 2008, 16, 5764–5769. 10.1364/OE.16.005764. 18542685

[ref37] Melissinaki V.; Farsari M.; Pissadakis S. A Fiber-Endface, Fabry–Perot Vapor Microsensor Fabricated by Multiphoton Polymerization. IEEE J. Sel. Top. Quantum Electron. 2015, 21, 344–353. 10.1109/JSTQE.2014.2381463.

[ref38] Zhang D.; Wei H.; Hu H.; Krishnaswamy S. Highly Sensitive Magnetic Field Microsensor Based on Direct Laser Writing of Fiber-Tip Optofluidic Fabry–Pérot Cavity. APL Photonics 2020, 5, 076112 10.1063/5.0012988.

[ref39] Zhao Y.; Wang P.; Lv R.; Liu X. Highly Sensitive Airflow Sensor Based on Fabry–Perot Interferometer and Vernier Effect. J. Lightwave Technol. 2016, 34, 5351–5356. 10.1109/JLT.2016.2615054.

[ref40] Williams J.; Suelzer J. S.; Usechak N. G.; Chandrahalim H. Optical Fiber Tip Micro Anemometer. 2020 IEEE Sensors 2020, 1–4. 10.1109/SENSORS47125.2020.9278804.

[ref41] Williams J.; Chandrahalim H. Optical Fiber Tip Micro Anemometer. U.S. Patent US20210341320A1, November 4, 2021.

[ref42] Liu G.; Sheng Q.; Hou W.; Han M. Optical Fiber Vector Flow Sensor Based on a Silicon Fabry–Perot Interferometer Array. Opt. Lett. 2016, 41, 4629–4632. 10.1364/OL.41.004629. 28005853

[ref43] Liu S.; Ji Y.; Yang J.; Sun W.; Li H. Nafion Film Temperature/Humidity Sensing Based on Optical Fiber Fabry-Perot Interference. Sens. Actuators, A 2018, 269, 313–321. 10.1016/j.sna.2017.11.034.

[ref44] Chen M.; Zhao Y.; Wei H.; Zhu C.; Krishnaswamy S. 3D Printed Castle Style Fabry-Perot Microcavity on Optical Fiber Tip as a Highly Sensitive Humidity Sensor. Sens. Actuators, B 2021, 328, 128981 10.1016/j.snb.2020.128981.

[ref45] Li M.; Liu Y.; Zhao X.; Gao R.; Li Y.; Qu S. High Sensitivity Fiber Acoustic Sensor Tip Working at 1550nm Fabricated by Two-Photon Polymerization Technique. Sens. Actuators, A 2017, 260, 29–34. 10.1016/j.sna.2017.03.040.

[ref46] Kilic O.; Digonnet M.; Kino G.; Solgaard O. External Fibre Fabry–Perot Acoustic Sensor Based on a Photonic-Crystal Mirror. Meas. Sci. Technol. 2007, 18, 3049–3054. 10.1088/0957-0233/18/10/S01.

[ref47] Wu S.; Wang L.; Chen X.; Zhou B. Flexible Optical Fiber Fabry–Perot Interferometer Based Acoustic and Mechanical Vibration Sensor. J. Lightwave Technol. 2018, 36, 2216–2221. 10.1109/JLT.2018.2810090.

[ref48] Thompson A. J.; Power M.; Yang G.-Z. Micro-Scale Fiber-Optic Force Sensor Fabricated Using Direct Laser Writing and Calibrated Using Machine Learning. Opt. Express 2018, 26, 14186–14200. 10.1364/OE.26.014186. 29877460

[ref49] Wen H.-Y.; Liu Y.-C.; Chiang C.-C. The Use of Doped Conductive Bionic Muscle Nanofibers in a Tennis Racket–Shaped Optical Fiber Humidity Sensor. Sens. Actuators, B 2020, 320, 128340 10.1016/j.snb.2020.128340.

[ref50] Power M.; Thompson A. J.; Anastasova S.; Yang G.-Z. A Monolithic Force-Sensitive 3D Microgripper Fabricated on the Tip of an Optical Fiber Using 2-Photon Polymerization. Small 2018, 14, 1703964 10.1002/smll.201703964. 29479810

[ref51] Gissibl T.; Thiele S.; Herkommer A.; Giessen H. Two-Photon Direct Laser Writing of Ultracompact Multi-Lens Objectives. Nat. Photon. 2016, 10, 554–560. 10.1038/nphoton.2016.121.

[ref52] Liu Q.; Zhan Y.; Zhang S.; Feng S.; Wang X.; Sun W.; Ye J.; Zhang Y. “Optical Tentacle” of Suspended Polymer Micro-Rings on a Multicore Fiber Facet for Vapor Sensing. Opt. Express 2020, 28, 11730–11741. 10.1364/OE.390145. 32403678

[ref53] Zhang S.; Tang S.-J.; Feng S.; Xiao Y.-F.; Cui W.; Wang X.; Sun W.; Ye J.; Han P.; Zhang X.; Zhang Y. High-Q Polymer Microcavities Integrated on a Multicore Fiber Facet for Vapor Sensing. Adv. Opt. Mater. 2019, 7, 1900602 10.1002/adom.201900602.

[ref54] Markiewicz K.; Wasylczyk P. Photonic-Chip-on-Tip: Compound Photonic Devices Fabricated on Optical Fibers. Opt. Express 2019, 27, 8440–8445. 10.1364/OE.27.008440. 31052661

[ref55] Hadibrata W.; Wei H.; Krishnaswamy S.; Aydin K. Inverse Design and 3D Printing of a Metalens on an Optical Fiber Tip for Direct Laser Lithography. Nano Lett. 2021, 21, 2422–2428. 10.1021/acs.nanolett.0c04463. 33720738

[ref56] Wang H.; Xie Z.; Zhang M.; Cui H.; He J.; Feng S.; Wang X.; Sun W.; Ye J.; Han P.; Zhang Y. A Miniaturized Optical Fiber Microphone with Concentric Nanorings Grating and Microsprings Structured Diaphragm. Opt. Laser Technol. 2016, 78, 110–115. 10.1016/j.optlastec.2015.08.009.

[ref57] Williams J. C.; Suelzer J. S.; Usechak N. G.; Chandrahalim H. In Optical Fiber-Tip Pressure Sensor Featuring a Spring Body and Multipositional Fabry–Pérot Cavity Resonator, 2021 IEEE 34th International Conference on Micro Electro Mechanical Systems (MEMS) 2021; pp 382–385.

[ref58] Williams J.; Chandrahalim H. Monolithically Integrated Microscale Pressure Sensor on an Optical Fiber Tip. U.S. Patent US20210325270A1, October 21, 2021.

[ref59] Ismail N.; Kores C. C.; Geskus D.; Pollnau M. Fabry-Pérot Resonator: Spectral Line Shapes, Generic and Related Airy Distributions, Linewidths, Finesses, and Performance at Low or Frequency-Dependent Reflectivity. Opt. Express 2016, 24, 16366–16389. 10.1364/OE.24.016366. 27464090

[ref60] Bitarafan M. H.; DeCorby R. G. On-Chip High-Finesse Fabry-Perot Microcavities for Optical Sensing and Quantum Information. Sensors 2017, 17, 1748 10.3390/s17081748. PMC5579499 28758967

[ref61] Anderson John D. Jr Fundamentals of Aerodynamics; 5th ed.; McGraw-Hill, 2010.

[ref62] Alzaatreh A. An Alternative to the Cauchy Distribution. MethodsX 2019, 6, 938–952. 10.1016/j.mex.2019.02.025. 31367528 PMC6650388

[ref63] Kilic O.; Digonnet M. J. F.; Kino G. S.; Solgaard O. Asymmetrical Spectral Response in Fiber Fabry–PÉrot Interferometers. J. Lightwave Technol. 2009, 27, 5648–5656. 10.1109/JLT.2009.2032135.

[ref64] Shaheen A. K.; Sabry Y. M.; Khalil D. Modeling of Fabry-Perot Micro Cavities Under Partial Spatial Coherence Illumination Using Multimode Optical Fibers. J. Lightwave Technol. 2021, 39, 4424–4430. 10.1109/JLT.2021.3069898.

[ref65] Ladner I. S.; Cullinan M. A.; Saha S. K. Tensile Properties of Polymer Nanowires Fabricated: Via Two-Photon Lithography. RSC Adv. 2019, 9, 28803–28813. 10.1039/c9ra02350j. PMC9071184 35529657

[ref66] Rohbeck N.; Ramachandramoorthy R.; Casari D.; Schürch P.; Edwards T. E. J.; Schilinsky L.; Philippe L.; Schwiedrzik J.; Michler J. Effect of High Strain Rates and Temperature on the Micromechanical Properties of 3D-Printed Polymer Structures Made by Two-Photon Lithography. Mater. Des. 2020, 195, 108977 10.1016/j.matdes.2020.108977.

[ref67] Bauer J.; Guell Izard A.; Zhang Y.; Baldacchini T.; Valdevit L. Programmable Mechanical Properties of Two-Photon Polymerized Materials: From Nanowires to Bulk. Adv. Mater. Technol. 2019, 4, 1900146 10.1002/admt.201900146.

[ref68] Lemma E. D.; Rizzi F.; Dattoma T.; Spagnolo B.; Sileo L.; Qualtieri A.; De Vittorio M.; Pisanello F. Mechanical Properties Tunability of Three-Dimensional Polymeric Structures in Two-Photon Lithography. IEEE Trans. Nanotechnol. 2017, 16, 23–31. 10.1109/TNANO.2016.2625820.

