# Supplementary material for: Multiphoton Nanosculpting of Optical Resonant and Nonresonant Microsensors on Fiber Tips
Source: ACS Appl Mater Interfaces. 2022 Apr 12;14(17):19988–99. doi: 10.1021/acsami.2c01033 (PMC9073840; doi:10.1021/acsami.2c01033)
Supplement: Supplementary file 1 — am2c01033_si_001.pdf [file am2c01033_si_001.pdf]

Supporting Information

Multiphoton nanosculpting of optical resonant and non-resonant microsensors on fiber tips

Jeremiah C. Williams,[†] Hengky Chandrahalim,[*,†] Joseph S. Suelzer,[‡] and Nicholas G. Usechak,[‡]

†*Department of Electrical and Computer Engineering, Air Force Institute of Technology, Wright–Patterson Air Force Base, Ohio 45433,USA*

‡*Sensors Directorate, Air Force Research Laboratory, Wright–Patterson Air Force Base, Ohio 45433, USA*

*E-mail: hengky@microsystems.group

A mechanically suspended hemispherical Fabry–Pérot cavity was fabricated using two-photon polymerization process as shown in Figure S1a. However, the inner optical surface of the cavity was shadowed by the top surface which prevented reflective coating deposition. Depositing a gold reflective film by sputtering improved the Q-factor of the Fabry–Pérot resonator as shown in Figure S1b. However, the measured spectrum (blue curve in Figure S1b) was consistent with resonance between the topmost and bottommost optical surfaces only, eliminating the optical response of the inner cavity.

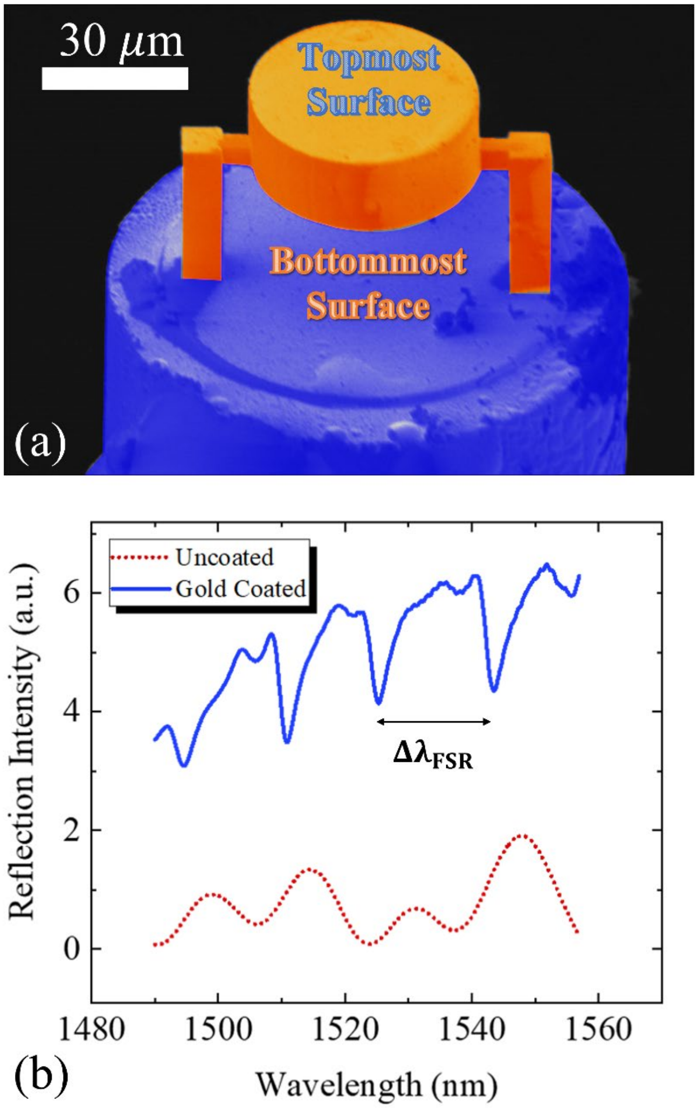

Figure S1. (a) A false-colored SEM image of a mechanically suspended hemispherical Fabry–Pérot cavity fabricated using two-photon polymerization nanofabrication process. (b) Measured reflection intensity before and after reflective coating deposition as a function of optical wavelength.

Although two-photon polymerization nanofabrication process is considerably faster than other nanofabrication techniques, the stepwise laser writing process presented striations into the surface finish of the fabricated structures. Hemispherical Fabry–Pérot cavities require a smooth spherical mirror, and it was uncertain if the devices created here had an optical-quality surface finish. Also of concern, features with a height equal to one half or one quarter of the wavelength of interest could introduce destructive interference and create an antireflective surface. To analyze the surface finish, we fabricated a sample structure onto a glass slide to mount into an atomic force microscope (AFM). The resultant AFM scan is shown in Figure S2.

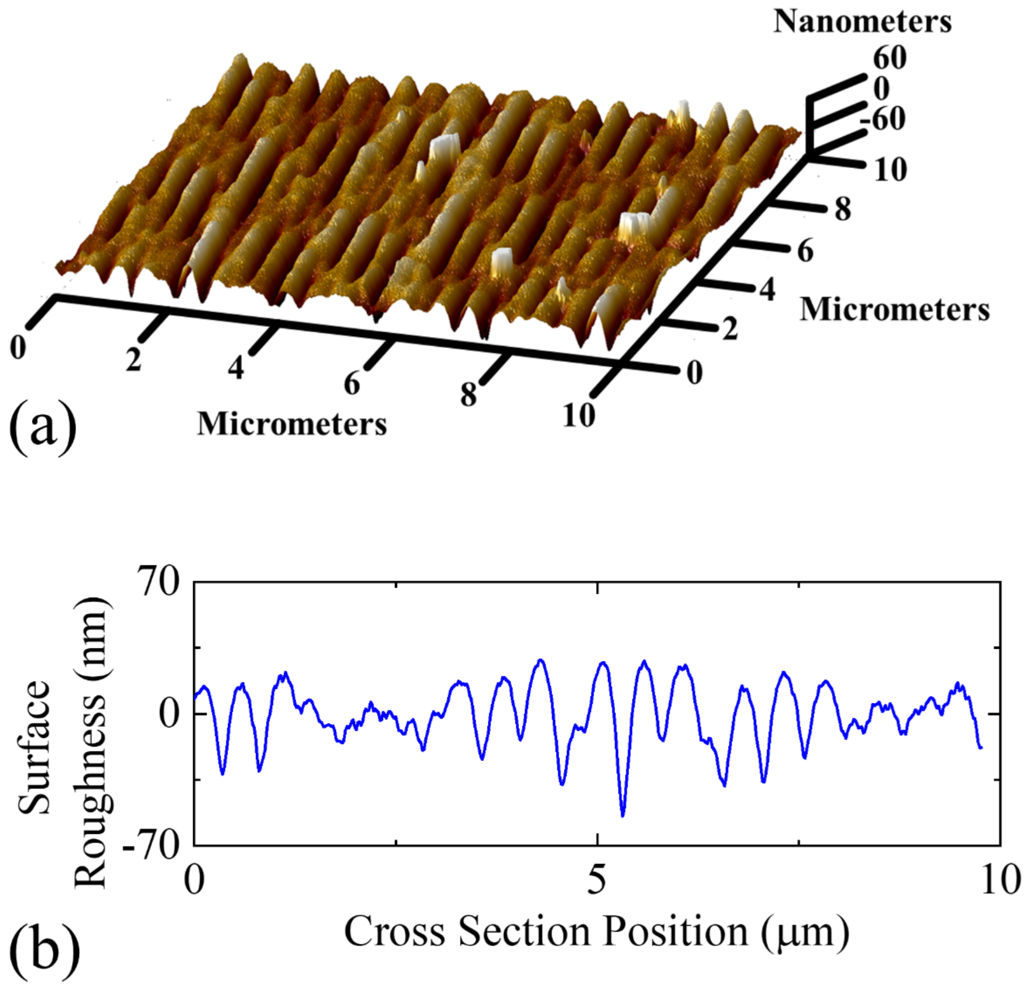

Figure S2. Surface analysis of an optical flat fabricated with two-photon polymerization by ultrashort laser pulses. a) A 3D rendering of the atomic force microscope (AFM) scan showing surface topography. b) A cross-sectional plot to quantify the surface roughness.

Research was performed to examine suitable highly reflective coating materials that can be deposited on the optical surfaces of the cavity to improve its reflectivity. Glass slides (76 mm x 24 mm x 1 mm) were coated on each side with thin film dielectrics and metals. Series of measurements to select a coating material that yields the highest optical Q-factor were performed according to the experimental setup in Figure S3a. Results plotted in Figure S3b suggest that 20 nm of gold deposited by a magnetron sputtering system should be used as a reflective coating material to enhance the Q-factor of the cavity.

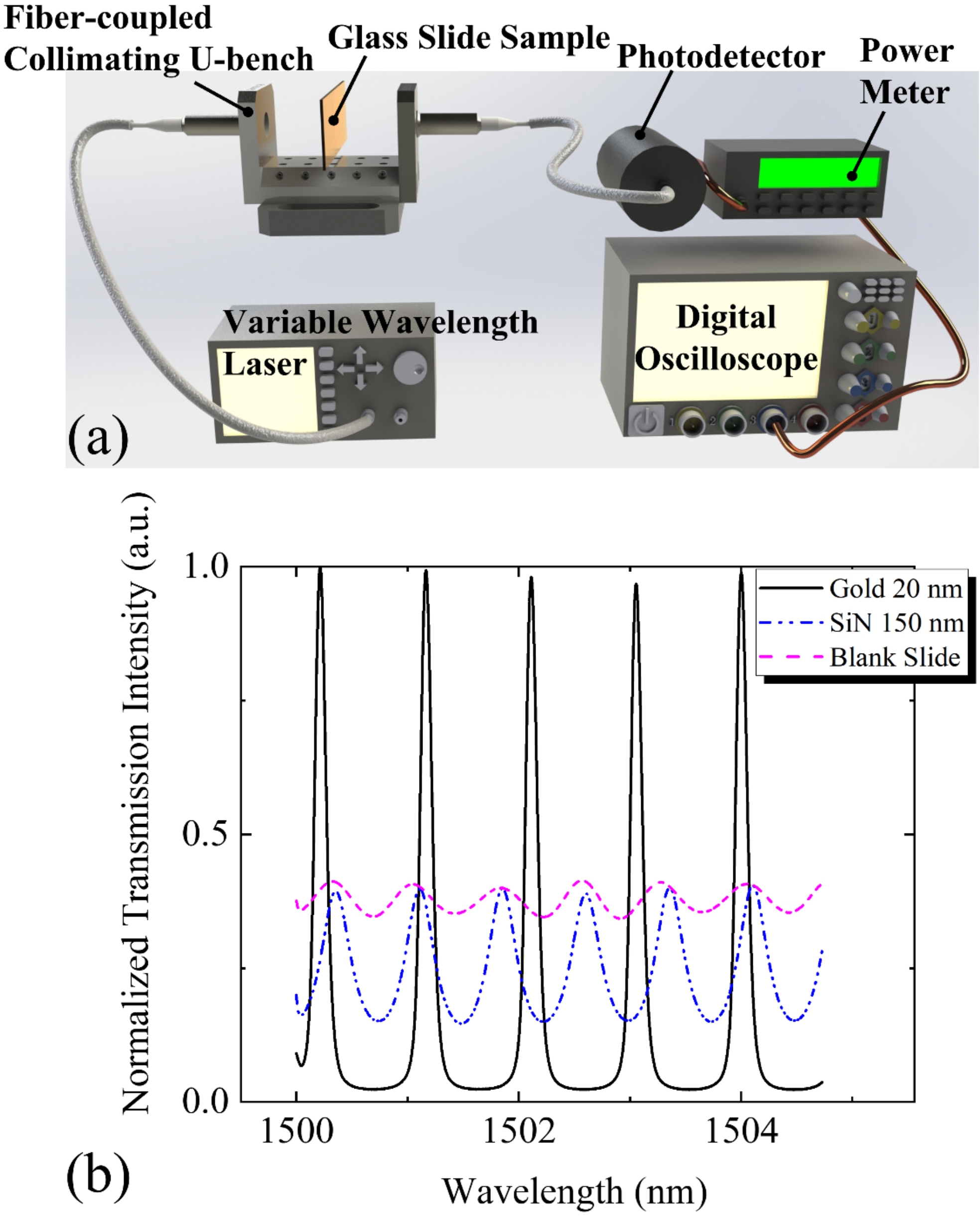

Figure S3. a) A schematic describing the measurement setup to select a coating material that yields the highest reflectivity. b) Measured transmission spectra through coated glass samples as a function of optical wavelength.

Video S1. Animation of the laser inscription process to create a spring body Fabry–Pérot cavity with rotatable top mirror on an optical fiber tip.

Video S2. Animation of the laser inscription process to create an optical non-resonant microturbine flow sensor on an optical fiber tip.

Video S3. A movie that shows a rotating flow sensor in response to incident nitrogen flow.

Table S1. Sputtering settings used to deposit the gold reflective coating for the spring body FPC.

Parameters	Values
Cathode Power	100 Watt
Cathode Mode	DC
Burn-in Time	30 seconds
Burn-in Pressure	10 mTorr
Deposition Pressure	5 mTorr
Stage Rotation	5 rpm
Fiber Position	Center Stage
Deposition Time	52 seconds

Table S2. Sputtering settings used to deposit the gold reflective coating for the microturbine flow sensor.

Parameters	Values
Cathode Power	100 Watt
Cathode Mode	DC
Burn-in Time	30 seconds
Burn-in Pressure	10 mTorr
Deposition Pressure	5 mTorr
Stage Rotation	5 rpm
Fiber Position	Center Stage
Deposition Time	300 seconds
